# Topological Organization of Metabolic Brain Networks in Pre-Chemotherapy Cancer with Depression: A Resting-State PET Study

**DOI:** 10.1371/journal.pone.0166049

**Published:** 2016-11-10

**Authors:** Lei Fang, Zhijun Yao, Jianping An, Xuejiao Chen, Yuanwei Xie, Hui Zhao, Junfeng Mao, Wangsheng Liang, Xiangxing Ma

**Affiliations:** 1 Department of radiology, Qilu Hospital, First Affiliated Hospital of Shandong University, 107 Cultural West Road, Jinan, 250012, Shandong Province, P.R.China; 2 PET/CT Center, Affiliated Lanzhou General Hospital of Lanzhou Military Area Command, 333 South Binhe Road, Lanzhou, 730050, Gansu Province, P.R.China; 3 School of Information Science and Engineering, Lanzhou University, Lanzhou, Gansu Province, 730000, P.R.China; 4 Nuclear Medicine Department, Affiliated Lanzhou General Hospital of Lanzhou Military Area Command, 333 South Binhe Road, Lanzhou, 730050, Gansu Province, P.R.China; Michigan State University, UNITED STATES

## Abstract

This study aimed to investigate the metabolic brain network and its relationship with depression symptoms using ^18^F-fluorodeoxyglucose positron emission tomography data in 78 pre-chemotherapy cancer patients with depression and 80 matched healthy subjects. Functional and structural imbalance or disruption of brain networks frequently occur following chemotherapy in cancer patients. However, few studies have focused on the topological organization of the metabolic brain network in cancer with depression, especially those without chemotherapy. The nodal and global parameters of the metabolic brain network were computed for cancer patients and healthy subjects. Significant decreases in metabolism were found in the frontal and temporal gyri in cancer patients compared with healthy subjects. Negative correlations between depression and metabolism were found predominantly in the inferior frontal and cuneus regions, whereas positive correlations were observed in several regions, primarily including the insula, hippocampus, amygdala, and middle temporal gyri. Furthermore, a higher clustering efficiency, longer path length, and fewer hubs were found in cancer patients compared with healthy subjects. The topological organization of the whole-brain metabolic networks may be disrupted in cancer. Finally, the present findings may provide a new avenue for exploring the neurobiological mechanism, which plays a key role in lessening the depression effects in pre-chemotherapy cancer patients.

## Introduction

A recent statistics indicated that new cancer cases nearly reached 4.29 million, and cancer deaths hit 2.81 million in China in 2015 [1]. Cancer, a genetic disorder characterized by high incidence and mortality, is associated with an increased risk of depression and altered functional brain network topology [2, 3]. Depression and similar symptoms frequently occur in cancer patients following diagnosis, seriously influence the cognitive function, and cause memory impairment [4]. Depression-related factors may contribute to the less optimal network topology in the metabolic brain network of cancer patients [3].

Evidence from previous neuroimaging studies has shown cancer-associated morphological changes, such as lower gray and white matter volume and density [5], decreased hippocampal volume [6], and functional abnormalities, involving memory difficulties [3] and cognitive deficit [7]. At the same time, other studies reported that cerebral changes and impaired cognitive functioning were induced by chemotherapy [5, 8], or even caused by depression after diagnosis [9]. Fundamentally, cancer cells are so powerful because they have the novel capability for limitless replication and evading apoptosis, suggesting that they occupy the endowment of adjacent healthy cells to complete immortalized progression [10]. So, investigating the metabolic brain network in cancer patients could bring new insights into the information regarding the neurophysiological mechanisms.

Positron emission tomography (PET) with ^18^F-fluorodeoxyglucose (FDG) has been widely used in oncology for providing metabolic information [11]. The ability of PET to track biomarkers with high sensitivity makes it a powerful tool for studying cancer staging, therapeutic response, and recurrence [12]. Resting-state PET techniques can also be applied to validate hypotheses concerning the changes in functional connectivity that occur in various kinds of diseases such as schizophrenia [13], Alzheimer’s disease [14], depression [15], diabetic patients [16] and normal aging [17]. However, it remains largely unknown whether cancer and/or chemotherapy alter the topological organization of metabolic brain networks using ^18^F-FDG PET data.

The present study investigated abnormalities in resting-state metabolic brain networks using graph analysis in pre-chemotherapy cancer patients characterized by depression relative to matched normal controls (NCs). By considering a range of cancer categories and removing the effects of chemotherapy, it was hypothesized that cancer patients would show altered small-world properties and topological architecture in the metabolic brain network. The study also explored the significant decrease in regional metabolism in cancer patients and the potential association between regional hypo-metabolism and severity of depression symptoms.

## Materials and Methods

### Ethical statement

All participants gave written informed consent at the time of enrollment for PET image scanning, according to the Declaration of Helsinki (1991). The whole study was specifically approved by the Institutional Review Boards at Affiliated Lanzhou General Hospital of Lanzhou Military Area Command.

### Subjects

Among the 78 eligible participants, the most frequently occurring type of carcinoma was lung cancer, which accounted for 14% of cancer survivors. Next were breast (12%) and cervical (9%), gastric (9%) and intestinal (7%), and lymphomas (7%). The remaining were other carcinomas. The volunteer samples in the present study were recruited between August 2014 and December 2015, and the participants’ privacy was protected. The cancer patients included 44 minimal depressions (score 1–13, mean value: 6; SD: 3), 16 mild depressions (score 14–19, mean value: 15; SD: 1) and 18 moderate/severe depressions (score 20–63, mean value: 26; SD: 6). The demographic and clinical characteristics of the subjects are listed in Table 1. The depression symptoms were assessed by Beck Depression Inventory (BDI)-II [18].

**Table 1:** Demographic and clinical characteristics of the subjects.

	NCs (*n* = 80)	Cancer (*n* = 78)	*P* value
Gender (male/female)	45/35	43/35	0.887^a^
Age (mean ± SD)	49 ± 7	51 ± 11	0.157^b^
BDI-II (mean ± SD)	--	13 ± 9	--

^a^The *P* value was obtained using the chi-square test.

^b^The *P* value was obtained using the *tt-*test.

NC, Normal controls; BDI-II, Beck Depression Inventory-II; SD, standard deviation.

The eligibility criteria for NCs were as follows: (a) nondepressed, (b) no diagnosis of psychiatric disorder or mental disease, (c) no clinical history of cancer. The inclusion criteria for cancer patients were as follows: (a) age 18 years or older at the time of diagnosis, (b) symptoms developed within 1 year, (c) ability to tolerate ^18^F-fluorodeoxyglucose injection, (d) well-informed about his or her own condition.

### PET Image acquisition and preprocessing

One hundred and fifty-eight FDG-PET images were obtained using a Siemens Biograph TruePoint 64 PET/CT (Siemens Healthcare, Erlangen, Germany) in three-dimensional mode. The integrated computed tomography (CT) system was a 64 -slice scanner. The acquisition of co-registered CT and PET images was performed in one session. All participants’ preparation rules were strictly followed. Each subject fasted for at least 6 h to keep the blood glucose level within the scope of 3.9–6.1 mmol/L. The scan was performed not only 40–60 min after intravenous injection of 3.7 MBq/kg (maximum dose 370 MBq) equivalent to 0.1 mCi/kg (maximum dose = 10 mCi) of 18F-FDG, but also in a dimly lit room with minimal auditory stimulation during both injection and PET scanning. 2-dimensional (2D) and 3D were the common mode in PET imaging acquisition [19]. In the 2D mode, the total scan time for whole-body PET scanning was 15–25 min. In the 3D mode, only 7–9 min scan time was necessary and enough to obtain an overview of the FDG uptake in the brain [20]. In this study, brain images were acquired for 7 min in 3D mode. The noncontrast CT scan was performed immediately prior to the PET scan using a multi-detector 64-slice spiral CT scanner. The CT data on the combined scanner were used for PET attenuation correction. The FDG-PET data were reconstructed with ordered subset expectation maximization iterative algorithm. All images were preprocessed using the Statistical Parametric Mapping software (SPM8, http://www.fil.ion.ucl.ac.uk/spm) running under Matlab 7.14 on the CentOS 6.5. For analyzing PET images, individual PET images were normalized to the standard image data.

### Construction of metabolic brain networks using graph theory

In this study, ^18^F-FDG uptake was measured using PET technology to assess the resting-state cerebral glucose metabolism rates as proxy for neuronal activity [21]. The whole brain was segmented into 90 regions (45 regions in each hemisphere), corresponding approximately to automated anatomical labeling (AAL) atlas [22], to construct a metabolic brain network. Linear regression analysis was performed to remove the effects of age and gender, followed by correcting raw metabolism to the normalized value. Then, metabolic connections were defined as statistical associations in normalized glucose metabolism between each possible pair of brain regions, resulting in a N × N (90 × 90) correlation matrix (R) for NC subjects and cancer patients separately, in which element R_ij_ means the Pearson correlation coefficient between regions i and j [23]. The brain network construction procedure has been previously described in researches about various diseases [24–26]. Sparsity (S) was introduced as the ratio between the number of existing edges (E) and all possible edges (*N*(*N*– 1)/2), to simplify connection matrix into binarized and undirected matrix A, in which A_ij_ was set to 1 if R_ij_ was higher than threshold, or else 0 [27]. Diagonal elements A_ii_ were defined as 0, representing no-edge regions. In the present study, 24% was chosen as fixed sparsity for ensuring a fully connected form of matrices of both groups. Such a sparsity can guarantee two networks having the same number of edges and minimize the false-positive edges in the meantime [28]. However, a comprehensive comparison of network properties can be performed for a range of sparsity (24% ≤ S ≤ 50%) with increments of 1% [29]. This scope of sparsity gives attention to both fully connected and too-random-to-display small-world properties, as suggested in a previous study [30].

### Measures of metabolic brain network

Four global network properties (clustering coefficient, characteristic path length, small-world attribute, and connectivity patterns) and one regional nodal property (betweenness centrality) were combined to investigate the topological architecture of the metabolic brain network in NC and cancer groups.

Previous studies presented descriptions about clustering coefficient and characteristic path length [3, 23, 25]. In this study, definitions and formulations were briefly presented as follows:

$$C_{i}=\frac{2E_{i}}{D_{i}(D_{i}-1)}$$ (1)

where *E*_*i*_ is the total number of edges in the subnetwork composed of the neighbors of node *i*, and *D*_*i*_ is the number of edges connected to node *i*. Clustering coefficient (*C*_*p*_) for a network (R) is the average *C*_*i*_ (Eq 1) of all nodes, providing a measure to estimate the ability of information segregation for R [31].

$$L_{i}=\frac{1}{N-1}\sum_{\begin{array}{l} j\in R\\ i\neq j \end{array}}\min\{d_{ij}\}$$ (2)

where min{d_ij_} means the minimum path length between node *i* and all the other nodes *j*. Characteristic path length (*L*_*p*_) of a network is the average *L*_*i*_ (Eq 2) over all pairs of nodes, and is used as an essential symbol representing its global information integration [8]. To evaluate the brain network for a small-world regime, 1000 random networks with matched degree distribution, number of nodes, and edges were generated, and the corresponding values of *C*_random_ and *L*_random_ were calculated. By combining γ(*C*_*p*_/*C*_random_) with λ(*L*_*p*_/*L*_random_), the information processing capability of the actual brain network at global and regional levels can be easily deduced, compared with random networks [27].

$$B_{i}=\sum_{i\neq j\neq k\in R}\frac{\sigma_{j,k}(i)}{\sigma_{j,k}}$$ (3)

The betweenness centrality (*B*_*i*_) of node *i* is defined as the fraction of minimum path length among all possible pairs of nodes via the prescribed node *i*. The rationale for *B*_*i*_ was simply that this study intended to capture the most influential nodes over the flow of information in the network. Hubs were considered as those nodes that had a high value (>2 ×mean) of *B*_*i*_ in the network [32, 33].

### Statistical analysis

Possible whole-brain metabolism differences between cancer patients and healthy subjects were evaluated on a voxel-by-voxel basis using two-sample *t* tests in SPM8 implemented in Matlab (The Mathworks Inc., Sherborn, MA,USA) [34]. To determine whether the between-group metabolism differences were reliable and significant, a false discovery rate (FDR) correction was performed at a *P* value of 0.01. To study the effect of depression on metabolism, the BDI score was correlated with average values of regional metabolism using Pearson correlation coefficient algorithm and corrected using FDR (*P* <0.01).

To compare the statistical significance of the overall inter-regional connectivity between networks, the connectivity coefficients were converted from *r* to z utilizing Fisher’s *z* transform because of non-normal distribution [35]. Besides, for between-group differences in *C*_*p*_, *L*_*p*_, and *B*_*i*_, a nonparametric permutation test with 1000 repetitions was used at each threshold [36]. In each repetition, all participants were mixed as one group and then randomly reassigned into two groups that had the same size as the original groups. Finally, network parameters in randomized groups were regarded as confidence intervals of permutation distribution under the null hypothesis. The actual between-group differences were used to determine whether the differences were significant [37].

## Results

### Identifying regions of abnormal glucose metabolism in cancer patients

**Fig 1:**
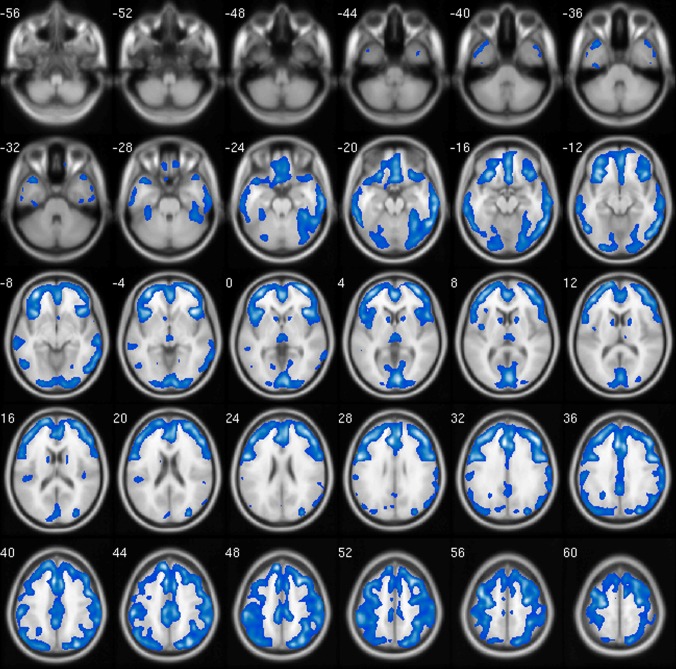
Differences in glucose metabolism between cancer patients and NC subjects. Blue represents hypo-metabolism in cancer patients compared with NC subjects. The results were calculated and displayed using the xjView (a viewing program for SPM, http://www.alivelearn.net/xjview8/).

The glucose metabolism values were first examined for each participant. Fig 1 shows the vertex-by-vertex metabolism differences between NC subjects and cancer patients. As expected, a significant decrease in metabolism was observed in cancer patients, indicating that the brain network of cancer patients had declining global efficiency or the brain regions had less neuronal activity. Table 2 presents detailed information about regions that showed significant lower glucose metabolism values in cancer patients, compared with NC subjects. The significance level for the clusters was set to be larger than 100 points and remained active after FDR correction at *P* <0.01.

**Table 2:** Brain regions demonstrating a significantly reduced glucose metabolism level in cancer patients.

AAL region	Cluster size	Peak (*T* value)	*X*	*Y*	*Z*
Temporal_Inf_R	149	–3.2082	37	3	–46
Frontal_Mid_L	23696	–6.2120	–4	28	31
Fusiform_L	2413	–3.8528	–34	–40	–27
Thalamus_R	3102	–4.2242	–3	–20	1
Caudate_L	1279	–3.5474	–11	8	7
Caudate_R	1188	–3.8244	17	8	10
Temporal_Sup_L	940	–3.6461	–50	–31	18
Insula	165	–3.0646	43	–26	16
Postcentral_R	724	–3.8455	10	–45	81
Frontal_Sup_L	127	–3.2631	–7	–12	80

*Note*: Cluster size is the number of voxels in the corresponding region. Peak (*T* value) is the maximum *t* value in the cluster. *X*, *Y*, and *Z* are the coordinates of peak vertex in Montreal Neurological Institute. Cluster size is obtained xjView8.14 toolbox (http://www.alivelearn.net/xjview) after multiple comparison correction (FDR, p<0.05).

### Correlation analysis between depression and metabolism

**Fig 2:**
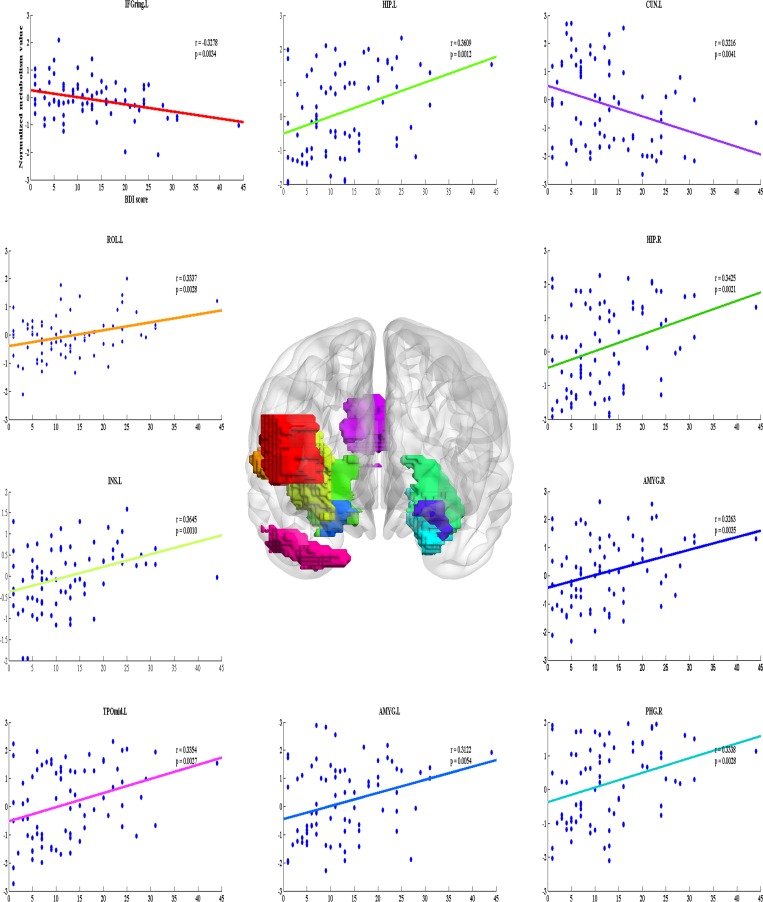
Brain regions displaying aberrant relationships between depression and metabolism in cancer patients. The regions were mapped onto the cortical surfaces and visualized using the BrainNet Viewer (http://www.nitrc.org/projects/bnv). Scatter plots with a curve-fitting line showing the metabolism values as a function of the depression state at minimum sparsity (S = 24%) in cancer patients. Abbreviation: IFGtriang.L = left Inferior frontal gyrus (triangular part); ROL.L = left Rolandic operculum; INS.L = left Insula; HIP.L = left Hippocampus; HIP.R = right Hippocampus; PHG.R = right Para-hippocampal; AMYG.L = left Amygdala; AMYG.R = right Amygdala; CUN.L = left Cuneus; TPOmid.L = left middle temporal gyrus. BDI-II, Beck Depression Inventory-II.

For the cancer patients, the BDI scores had a significant negative correlation with normalized metabolism levels in the left inferior frontal gyrus and cuneus. The curve-fitting line was downward sloping (*P* < 0.01), reflecting lower glucose metabolism values as measured by FDG-PET images and aggravated self-reported depressive symptoms indicated by the BDI scores in the left inferior frontal gyrus and cuneus at a fixed range of 24%. As illustrated in Fig 2, significant positive correlations were found between depression and metabolism in eight brain regions, mainly located in the parietal, limbic, and temporal lobes, such as the rolandic operculum, hippocampus, and para-hippocampus, which were involved in affective processing and mood regulation [38], and the insula and amygdala, which played a critical role in self-referential activity [39]. Negative correlations were observed in the triangular part of the inferior frontal gyrus and cuneus.

### Group differences in global network measures

**Fig 3:**
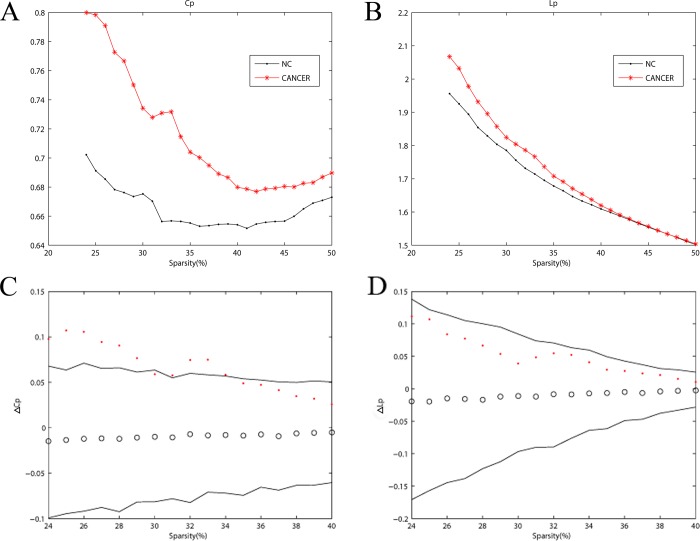
Cancer-related alterations in global network measures. (A) Clustering coefficient *C*_*p*_ and (B) characteristic path length *L*_*p*_ for the cancer (red stars) and the control group (black dot) as a function of sparsity S. (C) Comparison of the *C*_*p*_ and (D) *Lp* with counterparts of the corresponding random matrices with the same degree of distribution and the same number of edges and nodes. Black lines indicate the 95% confidence intervals, and the black hollow circles represent the mean values. The red dots represent Δ values, which equal *C*_*p*Cancer_−*C*_*p*NC_ or *L*_*p*_ Cancer−*L*_*p* NC_. No significant between-group differences in *L*_*p*_ were observed. Note that the small-world regime of sparsity in this study was from 24% to 40%.

Rather than limiting the analysis to a fixed network obtained using minimum sparsity to the Pearson correlation matrix, the clustering coefficient was calculated, which was the characteristic path length for each fully connected network over a range of thresholds in both NC subjects and cancer patients. The cancer networks had a higher clustering coefficient with a sharp decline, and a longer characteristic path length decreased toward its minimum value as sparsity increased (Fig 3A and 3B), compared with NC networks. Additionally, the C_p_ values in cancer patients were significantly higher than the values in NC subjects, as detected by 1000 non-parametric permutation test with matched random networks, but the *L*_*p*_ values were not (Fig 3C and 3D).

**Fig 4:**
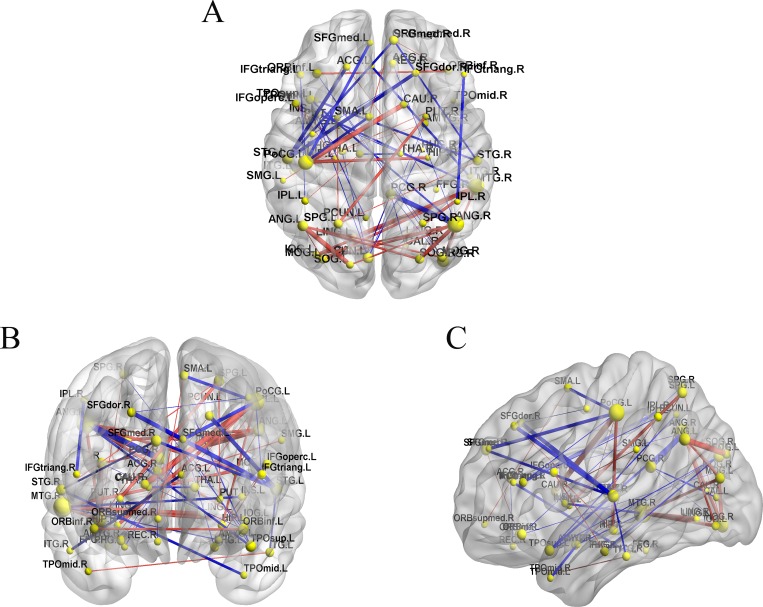
The significant difference connectivity map of resting-state brain metabolism networks between cancer and NC groups. (A) Axial, (B) coronal, and (C) sagittal. Connectivity increases (red lines) and decreases (blue lines) in cancer patients compared with NC subjects. Yellow spheres represent network regions, and the size is proportional to the number of times these regions are involved in a significant difference in connectivity (*P* < 0.01, uncorrected). Lines represent resting-state connectivity between regions, and the weight of the line indicates the strength of the difference in *Z* values.

The whole-brain topological organization can be revealed by analyzing the metabolism connectivity. Connectivity among brain regions provides an accurate quantitative analysis of the information parallel processing and transfer efficiency. The results of metabolism connectivity comparisons are presented in Fig 4, suggesting fundamentally different patterns in the metabolic brain network between cancer and NC group. It was found that the strength of connectivity was weakened in many brain regions in the parietal and frontal lobes in cancer patients, which predominantly belonged to long-range connectivity (Euclidean distance >75 mm). In contrast, the strength of short-range connectivity among the angular, postcentral, and occipital gyri and caudate area demonstrated an enhanced tendency (*P* = 0.01, uncorrected). These changes in connectivity might lead to regional efficiency aggrandizement and long path length disruption in cancer.

### Group differences in regional network measures

**Fig 5:**
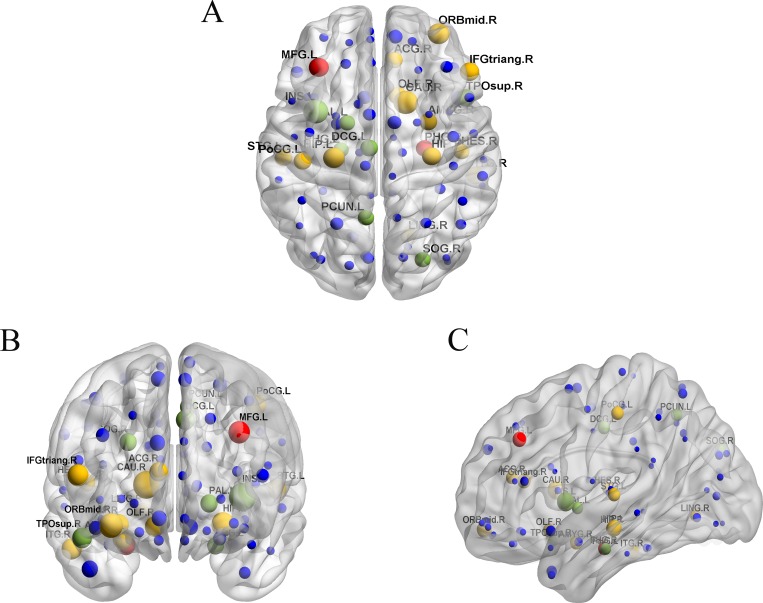
Network hubs in metabolic brain network. (A) Axial, B) coronal, and (C) sagittal. Metabolic correlation networks and hubs overlaid on the ICBM152 brain template. Hubs only in NC subjects are colored in yellow, hubs only in cancer patients are colored in green; hubs in both groups are colored in red, and nonhub regions are colored in blue. The size of the spheres is proportional to the betweenness value of nodes.

To elucidate the importance of hub regions for the brain network, several regions were identified as metabolism hubs in NC subjects. They are colored yellow in Fig 5, mainly located in the frontal, temporal and limbic lobes, such as the middle and inferior frontal gyri, anterior cingulate, superior and inferior temporal gyri, amygdala, and bilateral hippocampus regions. Green spheres in Fig 5 are hubs in cancer patients relative to the NC subjects for the insula, median cingulum, bilateral para-hippocampus, superior occipital gyrus, and superior temporal gyrus.

**Fig 6:**
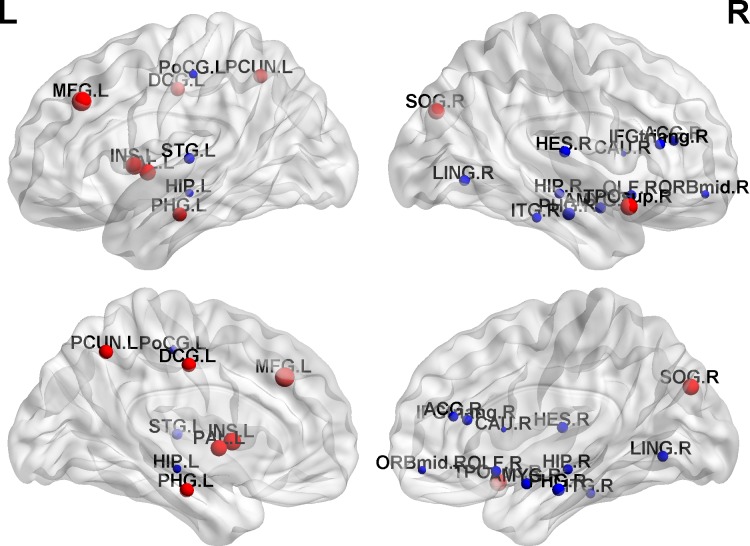
Brain regions showing abnormal nodal betweenness centralities in the metabolic brain network of cancer patients. Red nodes have significantly higher betweenness centralities, and the blue nodes have significantly lower betweenness centralities, in cancer patients. The size of the node is the absolute value of the differentials between cancer patients and NC subjects. L, left hemisphere; R, right hemisphere.

The GRETNA (https://www.nitrc.org/projects/gretna/) [40] was used to compute the betweenness centrality value of each node in the brain networks at minimum sparsity (S = 24%) for both groups. Compared with NC subjects, cancer patients displayed decreased nodal betweenness centralities in several regions of frontal, temporal, and limbic lobes, including the triangular part of the inferior frontal gyrus, the orbital part of the middle frontal gyrus, the olfactory cortex, the Heschl gyrus, the caudate nucleus, the bilateral hippocampus, the right para-hippocampus, the amygdala, and the anterior cingulate. Increased nodal betweenness centralities in cancer patients were mainly located in the middle frontal gyrus, insula, superior occipital gyrus, and pallidum (Fig 6).

## Discussion

In this study, graph-theoretic analysis based on FDG-PET imaging was used to investigate alterations of the metabolic brain networks in depressed cancer patients. The leading aim of this study was to explore the aberrant topological architecture of metabolic brain networks in cancer patients.

First, a comparison of whole-brain glucose uptake was performed to detect neuronal activity at region-level [41]. Then, the possible correlation between glucose metabolism abnormality and depression severity was further reviewed, and it was hypothesized that the impaired glucose utilization could result in disruption and dysfunction of the brain network in cancer patients. The differences in global and regional network properties coincided with neurodegeneration alterations in cancer patients treated with chemotherapy [42, 43].

### Regions of abnormal glucose metabolism in cancer

As expected, the decreased glucose metabolism values were quite widespread in cancer patients, particularly in the left middle frontal gyrus, right thalamus, and left fusiform regions (Fig 1). Hypo-metabolism in medial frontal gyrus (MFG) occurred in the left middle frontal gyrus correlated significantly with the severity of depression in AD patients [44], and the identically decreased metabolism was found in MFG in cancer patients (Table 2). Declining glucose uptake in the prefrontal gyrus was found to be associated with aggression and impulsion [45], which were prevalent in cancer patients [46]. Owing to abundance of fibers in the thalamus, fusiform gyrus, hippocampus, and amygdala [47], the decrease in betweenness centralities in the hippocampus and amygdala, as observed in the present study, might lead to lessened outflow to the thalamus and fusiform gyrus and subsequent hypometabolism in cancer patients [48].

### Correlation analysis between depression and metabolism in cancer

Recently, substantial evidence from systematic reviews using meta-analysis suggested that anxiety and depression were a concern in cancer patients [9, 49]. In order to explore the specific therapy, efforts should be made to locate the regions associated with depression and expound the linkage of depression with other factors such as glucose metabolism rate. The cancer patients showed a negative relationship between normalized glucose metabolism and depression in the triangular part of the inferior frontal gyrus and cuneus (Fig 2). Muller et al demonstrated dysregulation in the inferior frontal gyrus in depression patients [50]. Silverman et al found the decreased glucose metabolism in the inferior frontal gyrus in chemotherapy-treated cancer patients [51]. Significant hypo-metabolism was observed in the cuneus using FDG-PET in depression patients [52]. Previous research on depression using functional connectivity demonstrated that the amygdala, para-hippocampus, and hippocampus exhibited a high discriminative power in classification [53]. It was reported that regions with high sensitivity to negative emotion were hyperactive in processing negative information [54]. So, it was not surprising to find a significant positive correlation between normalized glucose metabolism and severity of depression in the amygdala, para-hippocampus, and hippocampus in cancer patients. The present findings were in agreement globally with the results of previous researches. That is, glucose metabolism abnormalities and depression symptoms might affect processing in the whole-brain network and thereby influence the social and emotional impairments in the daily life of cancer patients.

### Altered global brain network properties in cancer

Although the global and regional brain network properties in breast cancer have been reported in neuroimaging researches using functional magnetic resonance imaging [55] and PET [56], further clarification is still required, as a small number of patients enrolled and were category-specific in previous studies. Compared with NC subjects, the metabolic brain networks of cancer patients displayed less small-world attributes, with similar *L*_*p*_ (λ ≈ 1) but higher *C*_*p*_ (γ > 1) over a wide range of sparsity (Fig 3), reflecting reduced global integration and increased regional segregation ability consistent with previous researches [3, 42]. It is speculated that a compensation tuning mechanism exists, including changing the global pathway and adjusting regional activity to preserve a seesaw-like balance of the brain network.

It has been suggested that to a certain extent the local metabolic state reflects the region’s capability for interaction with other regions [57, 58]. Then, the between-group differences in the metabolism connectivity were examined to measure the capacity for information processing. In cancer patients, the decreased connectivity was mainly found in the postcentral region with the supplementary motor area [59] and superior frontal gyrus, compared with the counterparts in NC subjects. A diffusion-weighted imaging and fMRI study showed that the supplementary motor area and frontal regions could play an important role in conflict-induced slowing [60]. Previous data from neuroimaging showed that SMA was implicated in stopping movement action [61], the postcentral region was responsible for motor planning, and the superior frontal gyrus was in charge of self-awareness during sensorimotor processing [62]. The present results support the concept of an imbalance between expressing emotions and repressing impulsiveness processing in cancer [63]. The decreased connectivity with regions related to motor may result in the recruitment in the topological architecture of the brain network.

Although the occipital gyrus was not directly influenced in cancer, increased metabolism connectivity has been observed in the intra- and interoccipital regions. This overconnectivity pattern among the inferior and middle occipital gyri may suggest that visual function is particularly vulnerable in cancer. The present findings indicate that early abnormalities in metabolism connectivity, prior to the chemotherapy treatment, may serve as a biomarker of neurodegeneration associated with the visual regions of brain network. And visual dysfunctions (involving visual motor and visual scanning problems [64, 65], visual memory and visual learning declines [66, 67]) are the common deficits and contribute to the lower cognitive performance in cancer patients [67]. The caudate nucleus has been reported to play a pivotal role in motor processes, emotional reactivity, and executive functions in neuropsychological studies [68]. The enhanced metabolism connectivity between the caudate and postcentral gyri could be a result of the additional cost for regular motor performance in cancer patients. This is in line with preoperative magnetoencephalographic-based functional connectivity in brain gliomas, suggesting that increased functional connectivity is predictive of poorer neurological outcome following treatment [69].

### Altered regional brain network properties in cancer

As mentioned in the previous section, the hub region is an additional pivotal measurement for metabolic brain network organization. Two regions, the left middle frontal gyrus and the right para-hippocampus, were presented as hubs in both cancer patients and NC subjects (Fig 5). The middle frontal gyrus receives “What,” “When,” and “Where” information and has functional specialization in executive and decision-related processes [70]. Therefore, assigning an increased betweenness centrality to the middle frontal gyrus in the metabolic brain network in cancer patients may allude to poor control for emotion and movement integration. Cancer patients, prior to adjuvant treatment, likely have poor cognitive performance [71], and the para-hippocampal cortex is commonly thought to respond to emotional stimuli [72]. In accordance with the numerous previous researches, decreased betweenness centrality in the para-hippocampal cortex in the present study also showed an alteration in the regional properties in cancer patients.

The other hub regions in cancer mainly included the precuneus, median cingulate gyri, and insular cortex (Fig 6). Physical symptom burden, emotional pressure, and psychological torture result in a series of quality-of-life concerns for cancer patients [73]. PET imaging has provided evidence that the precuneus was linked with self-consciousness processes [74]. Critchley and his colleagues have proposed that higher gray matter volume and enhanced activity in insular and cingulate cortices were correlated with negative emotional experience using fMRI data [75]. In the present study, it was speculated that the increased betweenness centrality in the aforementioned three regions might have a hidden cost, suggesting difficulties in completing specialized function and poor daily performance [76]. This analysis of the hub regions reveals that impertinently adjusting to the disrupted brain network may be an effect immediately related to a lower neurobiological state, that is, weaker glucose metabolism and worse global efficiency.

## Conclusions

In summary, the present study quantitatively analyzed the characteristics of the metabolic brain network using FDG-PET imaging in cancer patients. Similar to NC subjects, cancer patients also preserved the small-world properties, but the topological architecture was found with partial loss of optimal pattern. The hypo-metabolism and its relationship with BDI scores may help explain the dysfunction and inefficiency of the metabolic brain network following the disease. The abnormalities in global and regional parameters were mainly observed in the frontal and temporal lobes as well as the limbic regions, which would be critical to the understanding of the neurobiological mechanism associated with depression symptoms in pre-chemotherapy cancer.

## Limitations

Several limitations need to be addressed in the present study. First, changes in the metabolic brain network focused on 90 regions of interest according to AAL atlas; thus, the subcortical and cerebellar regions still need to be studied. Another limitation was that years of education and hereditary factors were not included; these variables impact the risk of cancer [77, 78]. It is very important to examine how brain network changes in cancer are correlation with alterations of metabolic/anatomical/functional parameters, by combining sMRI, fMRI, DTI and PET data. Finally, the binary matrix used in the present study was unweighted and undirected. To fully understand the complex system of brain networks, a more precise matrix should be applied in the future.

## Supporting Information

S1 Table Abbreviations for regions in AAL atlas. (DOCX)
