# Supplementary material for: Topological Organization of Metabolic Brain Networks in Pre-Chemotherapy Cancer with Depression: A Resting-State PET Study
Source: PLoS One. 2016 Nov 10;11(11):e0166049. doi: 10.1371/journal.pone.0166049 (PMC5104370; doi:10.1371/journal.pone.0166049)
Supplement: S1 Table — (DOCX) [file pone.0166049.s001.docx]

**Supplement Material**

Table S1 Abbreviations for regions in AAL atlas.

| Labels | Regions | abbr. |
| --- | --- | --- |
| 1 | Precental gyrus | PreCG.L |
| 2 | Precental gyrus | PreCG.R |
| 3 | Superior frontal gyrus, dorsolateral | SFGdor.L |
| 4 | Superior frontal gyrus, dorsolateral | SFGdor.R |
| 5 | Superior frontal gyrus, orbital part | ORBsup.L |
| 6 | Superior frontal gyrus, orbital part | ORBsup.R |
| 7 | Middle frontal gyrus | MFG.L |
| 8 | Middle frontal gyrus | MFG.R |
| 9 | Middle frontal gyrus, orbital part | ORBmid.L |
| 10 | Middle frontal gyrus, orbital part | ORBmid.R |
| 11 | Inferior frontal gyrus, opercular part | IFGoperc.L |
| 12 | Inferior frontal gyrus, opercular part | IFGoperc.R |
| 13 | Inferior frontal gyrus, triangular part | IFGtriang.L |
| 14 | Inferior frontal gyrus, triangular part | IFGtriang.R |
| 15 | Inferior frontal gyrus, orbital part | ORBinf.L |
| 16 | Inferior frontal gyrus, orbital part | ORBinf.R |
| 17 | Rolandic operculum | ROL.L |
| 18 | Rolandic operculum | ROL.R |
| 19 | Supplementary motor area | SMA.L |
| 20 | Supplementary motor area | SMA.R |
| 21 | Olfactory cortex | OLF.L |
| 22 | Olfactory cortex | OLF.R |
| 23 | Superior frontal gyrus, medial | SFGmed.L |
| 24 | Superior frontal gyrus, medial | SFGmed.R |
| 25 | Superior frontal gyrus, medial orbital | ORBsupmed.L |
| 26 | Superior frontal gyrus, medial orbital | ORBsupmed.R |
| 27 | Gyrus rectus | REC.L |
| 28 | Gyrus rectus | REC.R |
| 29 | Insula | INS.L |
| 30 | Insula | INS.R |
| 31 | Anterior cingulate and paracingulate gyri | ACG.L |
| 32 | Anterior cingulate and paracingulate gyri | ACG.R |
| 33 | Median cingulate and paracingulate gyri | DCG.L |
| 34 | Median cingulate and paracingulate gyri | DCG.R |
| 35 | Posterior cingulate gyrus | PCG.L |
| 36 | Posterior cingulate gyrus | PCG.R |
| 37 | Hippocampus | HIP.L |
| 38 | Hippocampus | HIP.R |
| 39 | Parahippocampal gyrus | PHG.L |
| 40 | Parahippocampal gyrus | PHG.R |
| 41 | Amygdala | AMYG.L |
| 42 | Amygdala | AMYG.R |
| 43 | Calcarine fissure and surrounding cortex | CAL.L |
| 44 | Calcarine fissure and surrounding cortex | CAL.R |
| 45 | Cuneus | CUN.L |
| 46 | Cuneus | CUN.R |
| 47 | Lingual gyrus | LING.L |
| 48 | Lingual gyrus | LING.R |
| 49 | Superior occipital gyrus | SOG.L |
| 50 | Superior occipital gyrus | SOG.R |
| 51 | Middle occipital gyrus | MOG.L |
| 52 | Middle occipital gyrus | MOG.R |
| 53 | Inferior occipital gyrus | IOG.L |
| 54 | Inferior occipital gyrus | IOG.R |
| 55 | Fusiform gyrus | FFG.L |
| 56 | Fusiform gyrus | FFG.R |
| 57 | Postcentral gyrus | PoCG.L |
| 58 | Postcentral gyrus | PoCG.R |
| 59 | Superior parietal gyrus | SPG.L |
| 60 | Superior parietal gyrus | SPG.R |
| 61 | Inferior parietal, but supramarginal and angular gyri | IPL.L |
| 62 | Inferior parietal, but supramarginal and angular gyri | IPL.R |
| 63 | Supramarginal gyrus | SMG.L |
| 64 | Supramarginal gyrus | SMG.R |
| 65 | Angular gyrus | ANG.L |
| 66 | Angular gyrus | ANG.R |
| 67 | Precuneus | PCUN.L |
| 68 | Precuneus | PCUN.R |
| 69 | Paracentral lobule | PCL.L |
| 70 | Paracentral lobule | PCL.R |
| 71 | Caudate nucleus | CAU.L |
| 72 | Caudate nucleus | CAU.R |
| 73 | Lenticular nucleus, putamen | PUT.L |
| 74 | Lenticular nucleus, putamen | PUT.R |
| 75 | Lenticular nucleus, pallidum | PAL.L |
| 76 | Lenticular nucleus, pallidum | PAL.R |
| 77 | Thalamus | THA.L |
| 78 | Thalamus | THA.R |
| 79 | Heschl gyrus | HES.L |
| 80 | Heschl gyrus | HES.R |
| 81 | Superior temporal gyrus | STG.L |
| 82 | Superior temporal gyrus | STG.R |
| 83 | Temporal pole: superior temporal gyrus | TPOsup.L |
| 84 | Temporal pole: superior temporal gyrus | TPOsup.R |
| 85 | Middle temporal gyrus | MTG.L |
| 86 | Middle temporal gyrus | MTG.R |
| 87 | Temporal pole: middle temporal gyrus | TPOmid.L |
| 88 | Temporal pole: middle temporal gyrus | TPOmid.R |
| 89 | Inferior temporal gyrus | ITG.L |
| 90 | Inferior temporal gyrus | ITG.R |
